# Notification of expression of concern

**DOI:** 10.1111/aogs.14517

**Published:** 2023-02-16

Maher MA, Abdelaziz A, Ellaithy M, Bazeed MF. Prevention of preterm birth: a randomized trial of vaginal compared with intramuscular progesterone. Acta Obstet Gynecol Scand 2012; 92:215–222: DOI: 10.1111/aogs.12017

This note is for the above article, published online on 27 September 2012 in Wiley Online Library (wileyonlinelibrary.com), and has been published by agreement between the journal's Editor‐in‐Chief and John Wiley and Sons, Ltd. The note has been agreed due to concerns raised regarding the authenticity of the data presented within the article. The authors have provided a response to the journal regarding these allegations. The journal has requested an investigation from the author's institution, University of Menoufia, and an investigation is on‐going. As a result, the journal is issuing this note to readers. A subsequent note will be published depending on the outcome of the investigation.

