# Correction to “Notification: Prevention of preterm birth: a randomized trial of vaginal compared with intramuscular progesterone”

**DOI:** 10.1111/aogs.14616

**Published:** 2023-07-24

Maher MA, Abdelaziz A, Ellaithy M, Bazeed MF. Notification: Prevention of preterm birth: a randomized trial of vaginal compared with intramuscular progesterone. Acta Obstet Gynecol Scand 2023; https://doi.org/10.1111/aogs.14517.

In the above article, the display name has been changed from “Editorial Note” to “Notification” and the article title has been changed from “Notification of expression of Concern” to “Notification: Prevention of preterm birth: a randomized trial of vaginal compared with intramuscular progesterone”.

We apologize for this error.

